# Epicatechin and Catechin Modulate Endothelial Activation Induced by Platelets of Patients with Peripheral Artery Disease

**DOI:** 10.1155/2014/691015

**Published:** 2014-08-07

**Authors:** R. Carnevale, L. Loffredo, C. Nocella, S. Bartimoccia, T. Bucci, E. De Falco, M. Peruzzi, I. Chimenti, G. Biondi-Zoccai, P. Pignatelli, F. Violi, G. Frati

**Affiliations:** ^1^Department of Internal Medicine and Medical Specialties, Sapienza University of Rome, 00161 Rome, Italy; ^2^Department of Medical-Surgical Sciences and Biotechnologies, Sapienza University of Rome, Latina, Italy; ^3^Department of AngioCardioNeurology, IRCCS NeuroMed, 86077 Pozzilli, Italy

## Abstract

Platelet activation contributes to the alteration of endothelial function, a critical initial step in atherogenesis through the production and release of prooxidant mediators. There is uncertainty about the precise role of polyphenols in interaction between platelets and endothelial cells (ECs). We aimed to investigate whether polyphenols are able to reduce endothelial activation induced by activated platelets. First, we compared platelet activation and flow-mediated dilation (FMD) in 10 healthy subjects (HS) and 10 patients with peripheral artery disease (PAD). Then, we evaluated the effect of epicatechin plus catechin on platelet-HUVEC interaction by measuring soluble cell adhesion molecules (CAMs), NOx production, and eNOS phosphorylation (p-eNOS) in HUVEC. Compared to HS, PAD patients had enhanced platelet activation. Conversely, PAD patients had lower FMD than HS. Supernatant of activated platelets from PAD patients induced an increase of sCAMs release and a decrease of p-eNOS and nitric oxide (NO) bioavailability compared to unstimulated HUVEC. Coincubation of HUVEC, with supernatant of PAD platelets patients, pretreated with a scalar dose of the polyphenols, resulted in a decrease of sCAMs release and in an increase of p-eNOS and NO bioavailability. This study demonstrates that epicatechin plus catechin reduces endothelial activation induced by activated platelets.

## 1. Introduction

Atherosclerosis is a chronic vascular disease. Such a condition is not to be considered a disease in its own right but rather a process that contributes to the pathogenesis of several serious diseases. There is no selective agent responsible for the entire atherosclerotic process, which is considered the product of several concomitant interplays among different risk factors such as genetic predisposition, hyperlipidemia, diabetes mellitus, smoking, and hypertension [1]. Atherosclerosis develops progressively through continuous evolution of arterial wall lesions due to the accumulation of cholesterol-rich lipids and the associated inflammatory response [1].

It is clear that functional or morphological alterations in endothelial cells (ECs), platelets, and leukocytes appear to be critical in the evolution, progression, and clinical manifestation of the atherosclerosis [2]. Their interaction plays an important role and represents a key event, triggering and sustaining the inflammatory process in the arterial wall [2]. Platelets have a major role in vascular inflammation through the production and release of proinflammatory as well as prooxidant mediators, and by interacting with ECs. Such interplay between platelets and endothelium affects the entire course of development of atherosclerosis.

Platelets sustain the inflammatory process at all stages of atherosclerosis by expressing membrane molecules such as intercellular adhesion molecule-2 (ICAM-2), P-selectin, CD95L, and CD40L that regulate several biological functions in the vessel wall, including cellular adhesion and aggregation, chemotaxis, survival and differentiation, and angiogenesis [3, 4].

The most important damage to the endothelium is induced by reactive oxygen species (ROS) and prooxidant molecules, which are associated to various risk factors of atherosclerosis and produced by different cells, including platelets.

Vascular endothelium responds to mechanical and hormonal stimuli by releasing signaling molecules involved in the modulation of haemostasis, such as nitric oxide (NO) [5, 6], which acts by enabling mechanisms of relaxation and vasodilatation in vascular smooth muscle cells (VSMCs) and by exerting profound functional and morphological effects on the vascular wall. Nitric oxide also exerts important anti-inflammatory and antithrombotic effects [7]. In particular, ROS production rapidly inactivates the vasorelaxation molecule NO, contributing to endothelial dysfunction described as impaired vasodilatation [8].

In addition to the reduction in NO bioavailability, platelet-induced endothelium activation leads to increased expression of cell adhesion molecules (CAMs), such as intercellular adhesion molecule-1 (ICAM-1), vascular adhesion molecule-1 (VCAM-1), and E-selectin [9].

Among different clinical settings associated with atherosclerosis, peripheral artery disease (PAD) is considered* per se* a sign of systemic atherosclerosis. Moreover, PAD patients display endothelial dysfunction, as demonstrated by low flow-mediated dilatation (FMD) [10], which has become a conventional measure of endothelial dysfunction in human atherosclerosis [11]. Consistently with reduced FMD, PAD patients have enhanced NOX2 activation [12] and the catalytic subunit of NADPH oxidase, as well as the major enzyme generating ROS involved in the modulation of arterial tone [13, 14].

Epidemiological studies have shown that a diet rich in polyphenols is able to reduce cardiovascular events [15, 16]. Polyphenols, a heterogeneous group of molecules found primarily in fruits and vegetables along with their effects on platelets function, have been investigated by several authors. Among other activities, an increase in NO production associated with a decrease in ROS formation has been described [17, 18]. This finding is in accordance with our previous report indicating that polyphenols synergize in exerting antioxidant and antiplatelet effects [19]. In fact, we observed that epicatechin plus catechin is able to exert an antioxidant effect greater than individual polyphenols [19]. Nevertheless, few data concerning the effects of polyphenols on platelet-endothelium interaction are available in PAD patients. The aim of this study was to investigate whether polyphenols are able to modulate endothelial function via inhibition of platelet-endothelial interaction. Furthermore, we tested the effect of epicatechin plus catechin on the interaction between ECs and platelets.

## 2. Materials and Methods

### 2.1. Cross-Sectional Study

The study has been carried out in the outpatient clinic of our division between January 2013 and September 2013, on 10 patients, aged between 40 and 80 years, presenting with symptoms of intermittent claudication (for at least 6 months), and corresponding to Fontaine stage IIb. Ten consecutive subjects matched for age, gender, cardiovascular risk factors, and pharmacological treatments were enrolled in the same period as controls. Each PAD patient to be enrolled in the study had

1. claudication (defined as leg pain occurring after less than 200 meters of walking, relieved within 10 minutes after cessation of exercise, and being of presumed atherosclerotic origin);
2. ankle/brachial index (ABI), which was assessed as ankle/arm systolic blood pressure ratio by Doppler ultrasonography, lower than 0.90 on the worst leg at rest.

All subjects underwent a full medical anamnestic collection, physical examination, 12-lead ECG, laboratory tests, and measurement of ABI. Patients had to be in stable conditions without abrupt changes of ABI (>20%) in the last month before the enrolment. Exclusion criteria were represented by liver insufficiency, serious renal disorders (serum creatinine > 2.8 mg/dL), acute cerebrovascular disease, acute myocardial infarction, deep venous thrombosis, tabagism, and ongoing therapy with antioxidants. Written informed consent was obtained from all subjects; the study was conformed to the ethical guidelines of the 1975 Declaration of Helsinki and was approved by the Ethical Committee of Policlinico Umberto I, Sapienza University of Rome (ClinicalTrials.gov identifier: NCT01947712).

### 2.2. Platelet Preparation

Venous blood was drawn in trisodium citrate (3.8%, 1/10 (v : v)) from fasting HS and PAD patients who had not taken any drugs affecting platelet function for at least 14 days. To obtain platelet rich plasma (PRP), blood was centrifuged at 15 min at 180 g at room temperature and the supernatant PRP was separated. To avoid leukocyte contamination, only the top 75% of the PRP was collected. To obtain washed platelets, the PRP was washed with ACD (10/1 v/v) and suspended in fatty acid-free Tyrode's buffer (2 × 10^8^ platelets/mL, unless otherwise noted).

Washed platelets were treated with a scalar dose of epicatechin (0.1–10 *μ*M) plus catechin (0.1–10 *μ*M) and activated with arachidonic acid (AA) (0.5 mM) for 10 min at 37°C and supernatant was stored at −80°C unless otherwise indicated.

### 2.3. Human Umbilical Vein Endothelial Cells (HUVEC)

Human umbilical vein endothelial cells (HUVEC) were cultured as described [20]. Briefly, cells were expanded (2000 cells/cm^2^) in complete medium (EndoGRO-LS Complete Media Kit, Millipore). Cell morphology and growth were monitored by light microscopy and assessed by Trypan Blue (Sigma, St. Louis, USA). The culture was expanded until passage 5.

### 2.4. Platelet ROS Production

Cell suspension was incubated with 2′,7′-dichlorofluorescein diacetate (5 *μ*mol/L) for 15 minutes at 37°C. After incubation, platelets were activated with AA. Platelet ROS production was expressed as stimulation index (mean level of fluorescence in stimulated cells/mean level of fluorescence in unstimulated cells) (SI). Fluorescence intensity was analyzed on an Epics XL-MCL cytometer (Coulter Electronics) equipped with an argon laser at 510–550 nm (green). For every histogram, 50,000 platelets were counted to determine the proportion of positive platelets. The fluorescent signal generated by the probe was expressed as mean fluorescence intensity (SI). Intra-assay coefficient of variation was 5%.

### 2.5. Platelet 8-iso-PGF2*α* Assays

To measure isoprostanes formation, platelets were activated with AA. The supernatant was stored at −80°C until measurement. Quantification of isoprostanes was performed measuring 8-iso-PGF2*α* by a previously described and validated EIA assay method [21] and was evaluated* in vivo* and* in vitro*. Intra-assay and interassay coefficients of variation were 5.8% and 5.0%, respectively.

### 2.6. Platelet NOx

A colorimetric assay kit (Tema Ricerca, Italy) was used to determine the NO metabolites nitrite and nitrate (NOx) in 100 *μ*L of platelet or HUVEC suspension maintained under stirring condition for 10 min at 37°C. Intra-assay and interassay coefficients of variation were 2.9% and 1.7%, respectively.

### 2.7. Platelet H_2_O_2_ Concentration

A colorimetric assay kit (Tema Ricerca, Italy) was used to determine the H_2_O_2_ concentrationin 100 *μ*L of platelet suspension maintained under stirring condition for 10 min at 37°C. Intra-assay and interassay coefficients of variation were 2.1% and 3.7%, respectively.

### 2.8. ELISA Detection of Platelet sNOX2-dp

NOX2-derived peptide (NOX2-dp), a marker of NADPH oxidase activation, was detected by ELISA method as previously described by Pignatelli et al. [22]. The peptide was recognized by a specific monoclonal antibody against the amino acidic sequence (224–268 residues) of the extra membrane portion of NOX2. To measure sNOX2-dp, platelet suspension was activated with AA and 100 *μ*L of the supernatant stored at −80°C until measurement. Values were expressed as pg/mL; intra-assay and interassay coefficients of variation were 5.2% and 6%, respectively.

### 2.9. Detection of HUVEC sICAM1, sVCAM1, and sE-Selectin

A Custom Human 3-Plex Array (Tema Ricerca, Italy) was used to determine the sICAM1, sVCAM1, and sE-selectin concentration. Values were expressed as ng/mL; intra-assay and interassay coefficients of variation were 5.2% and 6%, respectively.

### 2.10. Western Blot Analysis of p-eNOS and Total eNOS

Equal amounts of protein (30 *μ*g/lane) estimated by Bradford assay were solubilized in a 2X Laemmli sample buffer containing 2-mercaptoethanol and loaded in a denaturing SDS/10% polyacrylamide gel. Western blot analysis was performed with monoclonal anti-mouse eNOS-PSer1177 (1 : 1000; BD Transduction Laboratory) (2 *μ*g/mL) and anti-mouse eNOS (1 : 1000; BD Transduction Laboratory) (2 *μ*g/mL) incubated overnight at 4°C. After incubation, the pure nitrocellulose membranes (0.45 *μ*m) were washed and incubated with goat anti-mouse IgG1-horseradish peroxidase for 2 hours. Immune complexes were detected by enhanced chemiluminescence. The developed spots were quantified by densitometric analysis on a NIH Image 1.62f analyzer, and the value was expressed as arbitrary units. Each sample was analyzed in triplicate. The results were expressed as P-eNOS/total eNOS ratio.

### 2.11. FMD and Carotid Intima-Media Thickness

Ultrasound assessment of FMD was investigated according to the recently reported guidelines [23] as previously described [24]. The coefficient of variation for FMD measurements, obtained on 3 separate occasions, was 12.5%. Longitudinal ultrasonographic scans of the carotid artery were obtained on the same day as the studies of the brachial artery reactivity and included the evaluation of the right and left common carotid arteries 1 cm proximal to the carotid bulb. FMD was performed with a 7.5 MHz linear-array transducer ultrasound system (Sonomed, Lake Success, NY).

### 2.12. *In Vitro* Study

*In vitro* study was performed in blood taken from 5 PAD patients and 5 HS (3 males and 2 females; mean age: 56 ± 6 years). We analysed the effect of scalar doses of epicatechin (0.1–10 *μ*M) plus catechin (0.1–10 *μ*M) on platelet-HUVEC interaction.

Only PAD patients platelets (2 × 10^8^/mL) were incubated for 30 min at 37°C with epicatechin (0.1–10 *μ*M) plus catechin (0.1–10 *μ*M) (mix) and stimulated for 10 min with AA (0.5 mM). After incubation, samples were immediately pelleted at 4°C, and the supernatants were added to semiconfluent endothelial monolayers with and without endothelial growth factor (EGF, 10 ng/mL).

After 60 minutes of coincubation, supernatants were removed by gentle washing, which was confirmed by light microscopy observation. Afterwards, cultures were left for a further period of 2 hours in incubator with basal medium and harvested by trypsin. Then, both HUVEC and HUVEC culture supernatants were collected for the analysis of sICAM1, sVCAM1, sE-selectin, NOx concentration, eNOS phosphorylation, and total eNOS.

### 2.13. Sample Size Determination

We computed the minimum sample size with respect to a two-tailed one-sample Student's *t*-test, considering (i) a difference for NOX to be detected between the healthy subjects and PAD patients as |*δ*| 15 *μ*M, (ii) homogeneous SDs between groups as 4, and (iii) type I error probability as *α* = 0.05 and power 1 − *β* = 0.90. This resulted in a minimum sample size of *n* = 4 for group. Additional patients and cases were included for a subset of experiments in order to provide more externally valid findings.

### 2.14. Statistical Analyses

Continuous variables are reported as mean ± standard deviation and were compared with Student's *t*-test. Categorical variables are reported as % and compared with the *χ* ^2^ test. Statistical significance was set at the 2-tailed 0.05 level. Computations were performed with SPSS 18 (IBM, Armonk, NY, USA).

## 3. Results

### 3.1. Cross-Sectional Study

Clinical characteristics of PAD patients and HS are reported in Table 1.

**Figure 1:**
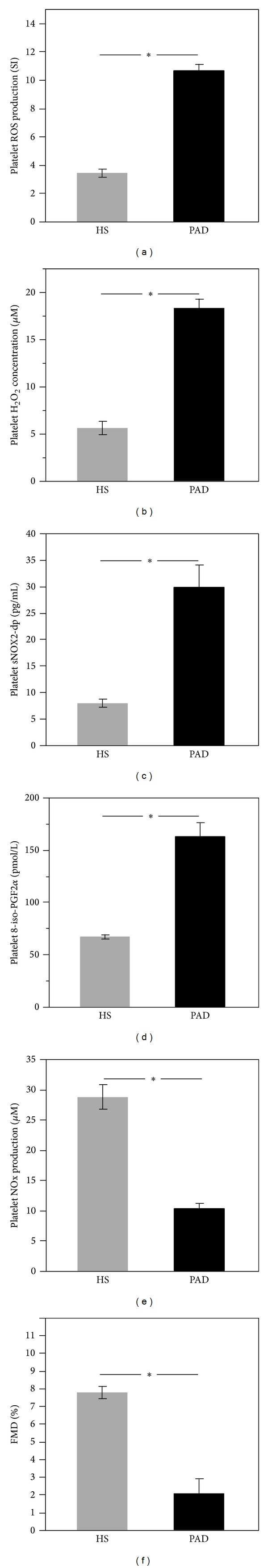
Platelet-induced oxidative stress in PAD patients. Platelet ROS production (a), platelet H_2_O_2_ concentration (b), platelet sNOX2-dp levels (c), platelet 8-iso-PGF2*α* production (d), platelet NOx production (e), and FMD (f) in PAD patients (*n* = 10) and healthy subjects (*n* = 10) ( *P < 0.001).

PAD patients showed higher platelet ROS production (10.6 ± 1.4 SI versus 3.4 ± 0.9 SI,  *P < 0.001) (Figure 1(a)), H_2_O_2_ concentration (18.4 ± 0.7 *μ*M versus 5.4 ± 0.5 *μ*M,  *P < 0.001) (Figure 1(b)), sNOX2-dp release (29.8 ± 13.4 pg/mL versus 7.9 ± 2.3 pg/mL,  *P < 0.001) (Figure 1(c)), and 8-iso-PGF2*α* production (162.6 ± 42.8 pmol/L versus 67.0 ± 6.4 pmol/L,  *P < 0.001) (Figure 1(d)) compared to HS. PAD patients displayed lower NOx production (28.7 ± 6.3 *μ*M versus 10.3 ± 2.6 *μ*M, *P* < 0.001) (Figure 1(e)) and FMD values (2.0 ± 2.5% versus 7.7 ± 1.0%,  *P < 0.001) (Figure 1(f)) compared with HS.

### 3.2. *In Vitro* Study

**Figure 2:**
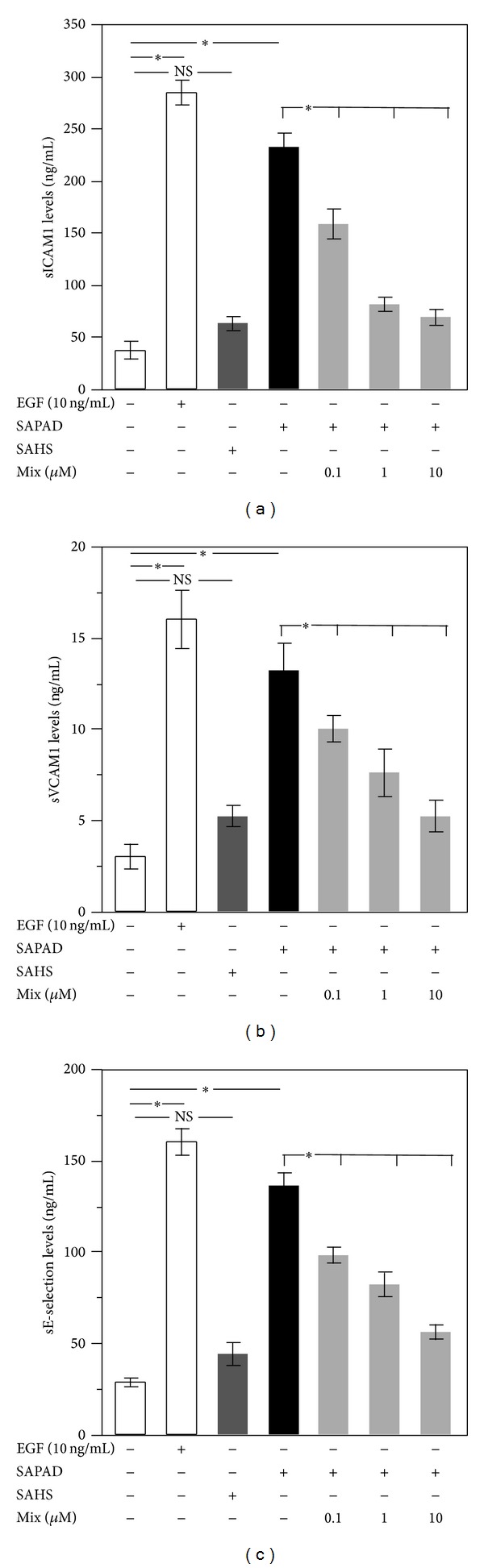
Biomarkers of HUVEC activation. sICAM1 (a), sVCAM1 (b), and E-selectin (c) levels released by HUVEC after incubation with EGF (white bar), supernatant of activated platelets from HS (SAHS) (dark gray bars), supernatant of activated PAD patients (SAPAD) (black bar), and supernatant of activated platelets from PAD patients (SAPAD), pretreated with scalar doses of epicatechin (0.1–10 *μ*M) plus catechin (0.1–10 *μ*M) (mix) (light gray bars) ( *P < 0.001).

HUVEC stimulated with EGF released a higher amount of sICAM1 (Figure 2(a),  *P < 0.001), sVCAM1 (Figure 2(b),  *P < 0.001), and E-selectin (Figure 2(c),  *P < 0.001) versus unstimulated HUVEC.

Supernatant of activated platelets from PAD patients (SAPAD) was able to increase sICAM1, sVCAM1, and sE-selectin versus nonstimulated HUVEC (Figure 2). Conversely, supernatant of activated platelets from HS (SAHS) was not able to change markers of endothelial dysfunction (Figure 2).

When SAPAD, pretreated with mix of epicatechin (0.1–10 *μ*M) and catechin (0.1–10 *μ*M), was added to HUVEC, a significant reduction of sICAM1, sVCAM1, and sE-selectin levels was observed compared to sCAMs levels induced by SAPAD (Figures 2(a), 2(b), and 2(c)).

**Figure 3:**
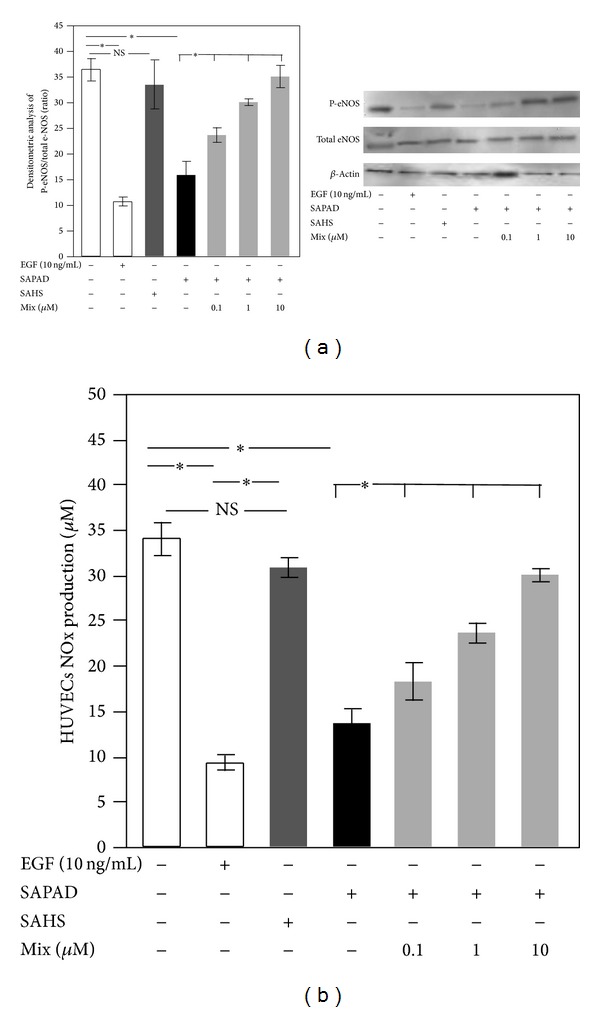
HUVEC eNOS activation and NOx production. Densitometric analysis of P-eNOS/total eNOS ratio (a) and NOx production (b) in HUVEC incubated with EGF (white bar), supernatant of activated platelets from HS (SAHS) (dark gray bars), supernatant of activated platelets from PAD patients (SAPAD) (black bar), and supernatant of activated platelets from PAD patients (SAPAD), pretreated with scalar doses of epicatechin (0.1–10 *μ*M) plus catechin (0.1–10 *μ*M) (mix) (light gray bars) ( *P < 0.001). A representative western blot analysis of eNOS phosphorylation (a).

Both the P-eNOS/total eNOS ratio (Figure 3(a),  *P < 0.001) and NOx concentration (Figure 3(b),  *P < 0.001) were decreased in HUVEC when stimulated with EGF. Moreover, we found that SAPAD contributed to the reduction of P-eNOS/total eNOS ratio compared to untreated HUVEC (Figure 3(a),  *P < 0.001) with consequent reduction of NO bioavailability (Figure 3(b),  *P < 0.0001). This phenomenon was not observed in HUVEC treated with SAHS (Figure 3).

When SAPAD, pretreated with mix of epicatechin (0.1–10 *μ*M) and catechin (0.1–10 *μ*M), was added to HUVEC, both the P-eNOS/total eNOS ratio and NO bioavailability increased compared to P-eNOS/total eNOS ratio and NO bioavailability induced by SAPAD (Figures 3(a) and 3(b)).

## 4. Discussion

The major findings of this paper are as follows: (a) PAD patients show higher platelet activation than HS; (b) supernatant of activated platelets derived from PAD patients (SAPAD) is able to activate ECs, resulting in an upregulation of soluble molecules derived from ECs (i.e., sICAM-1, sVCAM-1, and E-selectin); (c) PAD platelets induced ECs activation by reduction of p-eNOS and bioavailability of NO; (d) polyphenols are able to reduce endothelial activation induced by activated platelets.

These novel findings support the hypothesis that platelets-induced endothelial activation and thus the progression of atherosclerosis are a predisposing factor to more complex diseases such as PAD [25].

Previous findings showed that in PAD patients there is an imbalance between NOX2-mediated oxidative stress and FMD [11]. In the present study, we expanded such evidence demonstrating the key oxidative role of platelets in PAD patients by measuring their ROS production and sNOX2-dp release, as markers of NADPH oxidase activation [22], as well as their 8-iso-PGF2*α*-III production. We observed a significant increase of these oxidative stress markers in platelets of PAD patients compared to HS.

Activated platelets can interact with the endothelium triggering an inflammatory response that may contribute to the early stages of atherosclerosis [26]. Upon activation, platelets release granule contents able to activate ECs. It has been shown that activated platelets interact with the endothelium, inducing accumulation and transmigration of circulating monocytes, finally contributing to the atherosclerotic process at early stages [27]. Moreover, activated platelets can release in their microenvironment prooxidant molecules such as isoprostanes and ROS. In fact, we found that supernatant of PAD platelets is able to stimulate HUVEC, with consequent release of cellular adhesion molecules in their soluble form, namely, CAMs.

The soluble forms of adhesion molecules reflect endothelial CAM surface expression [28] and therefore can be used as biomarkers of endothelial activation in different diseases.

Other studies have shown that PAD patients have low FMD [24] strictly dependent on NO release [29]. In accordance with these findings, our PAD population had reduced FMD and lower nitrite and nitrate (markers of NO generation) compared to controls. NO is synthesized from the amino acid L-arginine by a family of enzymes known as nitric oxide synthase (NOS) [30]. Among three isoforms, eNOS is the major responsible for NO production in the cardiovascular system [31]. Enhanced eNOS inactivation, caused by excessive ROS production, may play an important role in the impairment of endothelium-dependent vasodilatation. When HUVEC were coincubated with SAPAD, a significant reduction in p-eNOS with consequent drop in NO bioavailability was observed.

In a previous study, we showed that the combination of epicatechin with catechin was able to boost the antioxidant and antiplatelet effects achieved by a single polyphenol [19]. Moreover, in a recent study, we observed that PAD patients treated with dark chocolate, rich in epicatechin and catechin, showed a decrease of oxidative stress 2 hours after its assumption [32].

In our study, we explored the hypothesis that polyphenols can downregulate platelets activation and subsequently reduce ECs activation. Therefore, we incubated HUVEC with supernatants of activated platelets pretreated with scalar doses of epicatechin plus catechin. These experiments showed that this mutual combination leads to a decrease in sCAM levels and an increase in eNOS phosphorylation with a higher bioavailability of NO.

The present study has implications and limitations that should be acknowledged. This paper suggests a potential role of polyphenols in the modulation of endothelial function but lacks a specific* in vivo* proof. Given that supernatants have been used in the study, it is possible to hypothesize by our results that platelets can exert their action by an indirect fashion. A deeper investigation on supernatant derived from PAD patients could highlight which specific molecules are responsible for these effects and their biochemical mechanism. Last, since ROS have a short half-life, thus representing a potential limitation of the study, a further step forward will be the evaluation of other mechanisms, independent from oxidative stress, or more stable markers of oxidative stress able to induce endothelial dysfunction. Besides, these results require further investigation in order to assess whether similar effects can be obtained* in vivo* by the administration of aliments or surrogates rich in polyphenols to PAD patients as in the hierarchy of events that underlie atherosclerotic remodeling of the adult human vessels, and the modulation of endothelial activation in PAD patients seems to play a key role.

In conclusion, this study demonstrates that polyphenols are able to modulate HUVEC activation induced by platelets derived from PAD patients.

## Figures and Tables

**Table 1:** Clinical characteristics of patients with peripheral artery disease (PAD) and healthy subjects (HS).

Variables	PAD (*n* = 10)	HS (*n* = 10)	*P* value
Mean age (year)^a^	63 ± 5	65 ± 6	0.854
Males/females	6/4	6/4	1.0
Hypertension % (*n*)^b^	98% (9)	0% (0)	<0.001
Diabetes mellitus % (*n*)	30% (3)	0% (0)	<0.001
Dyslipidemia % (*n*)	80% (8)	0% (0)	<0.001
Former smokers % (*n*)	40% (4)	50% (5)	0.621
CHD % (*n*)	20% (2)	0% (0)	<0.001
Glycemia (mg/dL)	108 ± 6	104 ± 4	0.589
Pharmacological treatments % (*n*)			
ACE-inhibitors	80% (8)	0% (0)	<0.001
Oral antidiabetic drugs	30% (3)	0% (0)	<0.001
Insulin	0% (0)	0% (0)	1.0
Statin	70% (7)	0% (0)	<0.001
Antiaggregants	60% (6)	0% (0)	<0.001
Oral anticoagulants	20% (2)	0% (0)	<0.001

^a^Data are expressed as mean ± SD.

^b^>139–89 mm/Hg.
